# Community engagement in outbreak response: lessons from the 2014–2016 Ebola outbreak in Sierra Leone

**DOI:** 10.1136/bmjgh-2019-002145

**Published:** 2020-08-23

**Authors:** Jamie Bedson, Mohamed F Jalloh, Danielle Pedi, Saiku Bah, Katharine Owen, Allan Oniba, Musa Sangarie, James S Fofanah, Mohammed B Jalloh, Paul Sengeh, Laura Skrip, Benjamin M Althouse, Laurent Hébert-Dufresne

**Affiliations:** 1 Restless Development Sierra Leone, Freetown, Sierra Leone; 2 Consultant to the Bill and Melinda Gates Foundation, Seattle, Washington, USA; 3 FOCUS 1000, Freetown, Sierra Leone; 4 Bill and Melinda Gates Foundation, Seattle, Washington, USA; 5 GOAL, Freetown, Sierra Leone; 6 BBC Media Action, London, UK; 7 Epidemiology, Institute for Disease Modeling, Bellevue, Washington, USA; 8 Information School, University of Washington, Seattle, Washington, USA; 9 Biology, New Mexico State University, Las Cruces, NM, United States; 10 Vermont Complex Systems Center, University of Vermont, Burlington, Vermont, USA; 11 Computer Science, University of Vermont, Burlington, Vermont, USA

**Keywords:** Community Engagement, Ebola, Sierra Leone

## Abstract

Documentation of structured community engagement initiatives and real-time monitoring of community engagement activities during large-scale epidemics is limited. To inform such initiatives, this paper analyses the Community Led Ebola Action (CLEA) approach implemented through the Social Mobilization Action Consortium (SMAC) during the 2014–2016 Ebola epidemic in Sierra Leone. The SMAC initiative consisted of a network of 2466 community mobilisers, >6000 religious leaders and 42 local radio stations across all 14 districts of Sierra Leone. Community mobilisers were active in nearly 70% of all communities across the country using the CLEA approach to facilitate community analysis, trigger collective action planning and maintain community action plans over time. CLEA was complemented by interactive radio programming and intensified religious leader engagement.

Community mobilisers trained in the CLEA approach used participatory methods, comprised of an initial community ‘triggering’ event, action plan development and weekly follow-ups to monitor progress on identified action items. Mobilisers collected operational and behavioural data on a weekly basis as part of CLEA. We conducted a retrospective analysis of >50 000 weekly reports from approximately 12 000 communities from December 2014 to September 2015. The data showed that 100% of the communities that were engaged had one or more action plans in place. Out of the 63 110 cumulative action points monitored by community mobilisers, 92% were marked as ‘in-progress’ (85%) or ‘achieved’ (7%) within 9 months. A qualitative examination of action points revealed that the in-progress status was indicative of the long-term sustainability of most action points (eg, continuous monitoring of visitors into the community) versus one-off action items that were marked as achieved (eg, initial installation of handwashing station). Analysis of behavioural outcomes of the intervention indicate an increase over time in the fraction of reported safe burials and fraction of reported cases referred for medical care within 24 hours of symptom onset in the communities that were engaged.

Through CLEA, we have demonstrated how large-scale, coordinated community engagement interventions can be achieved and monitored in real-time during future Ebola epidemics and other similar epidemics. The SMAC initiative provides a practical model for the design, implementation and monitoring of community engagement, integration and coordination of community engagement interventions with other health emergency response pillars, and adaptive strategies for large-scale community-based operational data collection.

**Summary box**

- Reviews of the 2014–2016 Ebola epidemic in West Africa identified community engagement and social mobilisation programmes as a critical component of the response and important contributing factor to ending transmission.
- The Social Mobilization Action Consortium (SMAC) was the largest coordinated community engagement initiative during the Sierra Leone Ebola outbreak, reaching more than 12 000 communities through 2466 trained community mobilisers, a network of 2000 mosques and churches and 42 local radio stations.
- We present the SMAC’s Community Led Ebola Action (CLEA) data set and undertake a retrospective analysis of the CLEA triggering and community action planning process and reported behavioural outcomes in engaged communities.
- The findings demonstrate that large-scale participatory community engagement and real-time data collection, including community-generated surveillance data on Ebola-safe behaviours, sickness and death, are achievable in the context of a health emergency if adequately structured, managed, coordinated and resourced.

**Summary box**

- The findings suggest lessons and innovations for large-scale community engagement that should be considered in future epidemics and other health emergencies. These include the need for: (1) recognition of community agency, two-way communications and active roles for communities in epidemic response; (2) prioritisation of community engagement interventions as a critical pillar of epidemic preparedness and response; (3) a supportive infrastructure for mobilisers and front-line workers; (4) standards of practice to guide quality and coordination of community engagement interventions; (5) integration of community engagement activities and data with other bio-medical pillars of disease response, in particular surveillance; (6) prioritisation of real-time community data collection and analysis to inform response decision-making; and (7) taking into account the imperatives of emergency response when defining ‘community’.

## Introduction

Community engagement and other community-centred approaches during public health emergencies are increasingly recognised as important components of health emergency preparedness and response, in order to foster enabling and reinforcing conditions for behaviour change to reduce the spread of disease.^1–3^ In 2009, the WHO convened a consultation to develop standards and identify best practices for community engagement in public health emergencies.^4^ The consultation concluded that there was a general under-appreciation of the behavioural imperative that underlies responses to public health emergencies, despite the fact that human behaviour drives epidemic emergence, transmission, and amplification. An interagency guide on communication for behavioural impact during an outbreak response was then developed by WHO, UNICEF and partners in 2012.^5^ Since then, recognition of the critical role of community engagement in a disease response has been reflected in a range of international guidelines and agreements.^6–9^

The importance of community engagement was exemplified during the 2014–2016 outbreak of Ebola Virus Disease (Ebola) in West Africa.^2 3^ Over the course of this outbreak, at least 28 616 cases occurred across Guinea, Sierra Leone, and Liberia.^10^ Sierra Leone alone accounted for 14 124 cases and 3956 deaths attributed to the Ebola outbreak.^10^ As numbers of cases rapidly increased, there was a growing consensus that large-scale behaviour change was required to reduce complex transmission risks posed by traditional burial and caregiving practices. Despite the availability of pre-existing behavioural guidelines, the operationalisation of integrated social mobilisation and community engagement interventions in Sierra Leone was challenged by insufficient capacity.^11^ In the context of an already fragile health system, the Ebola outbreak undoubtedly introduced new and unique challenges that the country was ill-prepared to handle.^12^

Early messaging overly emphasised Ebola as a ‘killer disease’ but fell short in providing actionable information on prevention, treatment, and possible survival.^12^ Initial emphasis on fear, as well as a lack of sensitivity to community values and traditions, contributed to people hiding from authorities and failing to seek medical care.^13^ This reflected experiences from previous outbreaks in Africa.^14^ At the same time, early anthropological research in Sierra Leone found that communities were willing to change behaviours and accept response measures such as safe burials if they were appropriately and continuously engaged.^15–17^ In August 2014, a national assessment of public knowledge, attitudes and practices found that Ebola awareness and knowledge were already high in Sierra Leone; however, misconceptions, stigma, and other barriers were prevalent.^13^ To address these issues, there was a need to develop systems for two-way communication and building linkages between demand-side activities and supply-side services.^11^

It is within this context that five partner organisations—GOAL (an international humanitarian response agency), Restless Development Sierra Leone (an international development agency), FOCUS 1000 (a Sierra Leonean non-governmental organisation), BBC Media Action (an international development charity), and the US Centers for Disease Control and Prevention—developed an integrated, community-led, data-driven approach to Ebola social mobilisation, with its core component consisting of a large-scale community engagement to support outbreak containment. The Social Mobilization Action Consortium (SMAC) was established in September 2014 and became operational in October 2014 in support of the Sierra Leone Ministry of Health and Sanitation’s Social Mobilization Pillar.

Previous research on community behaviour and practice in West Africa during the Ebola outbreak has found that communities have the capacity to rapidly acquire new knowledge and make change, but that effective behaviour change or adoption of safe practices can only occur when practical or realistic actions are in place to facilitate them.^17^ In addition, it has been documented that communities were able to develop and maintain local innovations in addressing Ebola risk.^15–18^ These findings reflect the understanding of the role of communities and the theory of change underpinning the design of the SMAC initiative.

In this paper, we describe SMAC’s approach to community engagement within the Sierra Leone outbreak response. We analyse over 50 000 semi-structured weekly reports from the network of SMAC community mobilisers (mobilisers). We draw upon this extensive data, and collective implementation experience, to identify key lessons and make recommendations for future design, implementation and research of community engagement activities within epidemic response and other health emergencies.

## Community engagement at scale: the CLEA approach

Restless Development and GOAL trained and supported nearly 2500 mobilisers who worked with communities to design and implement community action planning. FOCUS 1000 trained, engaged and supported over 6000 religious leaders from over 2000 mosques and churches to promote key messages and role model promoted behaviours, especially around safe burials. BBC Media Action supported 42 local radio station in all 14 districts to improve the quality and synchronisation of radio programming. Mobilisers were deployed in approximately 70% of communities in Sierra Leone across all 14 districts. Community engagement activities were complemented by near universal radio coverage and religious leader engagement (online supplementary information).

10.1136/bmjgh-2019-002145.supp1 Supplementary data

Mobilisers were recruited from an existing cohort of community health workers, former Restless Development youth volunteers and trusted people nominated by communities. Ebola survivors were also actively recruited as mobilisers, bringing with them first-hand experience of engaging with response mechanisms. This strategy of recruiting Ebola survivors also responded to the need for providing survivors with much-needed employment.^19^

Community engagement was facilitated through the Community-led Ebola Action (CLEA) approach (see table 1 and online supplementary information).^20^ CLEA draws on Participatory Learning and Action (PLA) programming in HIV/AIDS contexts^21 22^ and Community-Led Total Sanitation.^23^ It is also reflective of context-sensitive structured community engagement strategies used by Restless Development Sierra Leone’s pre-existing Volunteer Peer Education Programme. The CLEA approach was underpinned by the National Communications Strategy for Ebola Response in Sierra Leone developed in collaboration between the Ministry of Health and Social Mobilization Pillar partners,^24^ while the implementation of CLEA was regularly adjusted in response to findings of knowledge, attitudes, and practices assessments.^13^

**Table 1:** Comparison between health awareness and CLEA approaches

	Health education approaches	CLEA approaches
Unit of analysis	Individuals	Communities
Core activities	Educating households Sharing information and key messages	Listening to communities Inspiring self-realisation and self-motivated action
Communications approach	One-way information sharing Health educators as experts	Facilitating dialogue Community members as experts
Emphasis	Top-down Sharing biomedical facts, correcting erroneous beliefs	Bottom-up Appreciative of other ways of understanding illness Allow multiple framings for disease at the same time
Facilitation style	Teaching and preaching House-to-house	Listening and learning Community-wide
Methods and tools	Information, education and communication materials Lists of ‘Do’s’ and ‘Don’ts’	Participatory rural appraisal tools for communities Data collection that feeds back into approach
Typical assumptions	Traditional beliefs are the problem to be solved Communities must be convinced to use health services	Community responses can lower or enhance health Services must adapt to meet community needs
Key motivations for change	Awareness of biomedical facts Rational understanding of transmission routes Self-preservation	Urgency to protect each other, build on solidarity Build hope with early treatment Build trust in health authorities
Desired outcomes	Individuals seek external health services and follow the rules.	Communities feel empowered to protect themselves using local resources. Two-way dialogue results in better use of health services that respond to community needs.

Source: SMAC (2014), Community-led Ebola Action.^20^

CLEA, Community Led Ebola Action; SMAC, Social Mobilization Action Consortium.

Mobilisers completed a 1 week hands-on training programme, and subsequent refresher trainings, action-learning and other capacity building activities as part of their preparation for implementing the CLEA approach.^20^ Mobilisers received a monthly stipend (375 000 Sierra Leonean leone, approximately US$89. Minimum wage in Sierra Leone in 2014/2015 was 500 000 SLL) and support for transportation, communication, safety, and security and insurance. Rather than an ‘incentive’ to act, stipends were considered compensation for labour undertaken by mobilisers. This was complemented with comprehensive support and supervision. The CLEA approach and operational framework influenced best practices contained within the Standard Operating Procedures (SOPs) established to guide social mobilisation in Sierra Leone.^11 25^

A critique of the Ebola response in West Africa, especially in the early stages, was the emphasis on one-way, health communication messaging focussed on the disease itself.^26 27^ CLEA departed from one-way health education, communication and ‘messaging’ in two primary ways: (i) by using an interactive and iterative community facilitation approach comprised of ‘triggering events’ and development of community action plan. This approach facilitated communities to undertake their own appraisal and analysis of the Ebola outbreak, its current effects and the likely future impacts if no action is taken; (ii) operationalisation of a national, systematic feedback mechanism connecting communities through regular follow-up visits, access to dedicated mobile phone ‘closed user group’ and 24 hour mobiliser support. As a result, the CLEA model was focussed not only on supporting and encouraging Ebola-safe behaviour, but also providing a reliable communications infrastructure for directly linking large numbers of communities, via mobilisers and SMAC staff, with response authorities.

The aim of CLEA triggering events was to create a sense of urgency, a desire to act and local ownership. In each community, an initial triggering event was held using a set of six tailored PLA tools and facilitated by trained mobilisers. Communities received no payments for attendance and participation; however, community leaders received a comprehensive briefing on the programmes and its activities ahead of engagement. The participatory triggering activities consisted of: (i) Body Mapping; (ii) Danger Discussion; (iii) Burial Roleplay; (iv) Personal Protective Equipment Demonstration; (v) Ebola Survivor Stories; (vi) Ebola Spread Exercise. These are detailed in the CLEA manual.^20^ During the triggering event, facilitated group conversations and exercises were conducted to help community members undertake their own self-appraisal and analysis. For example, ‘Body Mapping’ and ‘Danger Discussion’ activities were used to visually represent and discuss community perceptions of Ebola symptoms, transmission and risk, and to discuss and rank individual and community ‘danger’ and what action may reduce these. ‘Burial Roleplay’ was used to explore understandings of what a typical burial in the community might entail, along with experience of dealing with burial response teams. Triggering events were conducted with the objective of developing community action plans and identification of ‘Community Champions’, typically influential community members who acted as focal points for programme activities.

Mobiliser training emphasised *community* identification of priority actions for action plans once an ‘ignition moment’ had been achieved and communities were receptive and prepared to develop an action plan. Mobilisers were trained with a set of indicative actions covering focus areas such as burials, reporting symptoms/deaths, reintegration of survivors and child protection, but also to anticipate that community priorities for action may include a combination of both Ebola-specific actions and non-Ebola-specific community priorities (an analysis of qualitative data collected by SMAC demonstrates the correlation between community-reported achievement and perception of a variety of priorities, such as autonomy and mastery, and Ebola-safe behaviour^28^). Action plans, often in the form of bylaws (such as restricting entrance to, and exit from, a community), were then implemented by communities and supported by Community Champions. Some communities explicitly included ‘repercussions’ as part of their bylaws, such as fines for non-compliance with visitor rules.

Mobilisers implemented the CLEA approach in both urban and rural areas. However, in the urban Western Area, the cohort of mobilisers also collected data during periodic house-to-house campaigns led by the Government of Sierra Leone using the same tool.

**Figure 1:**
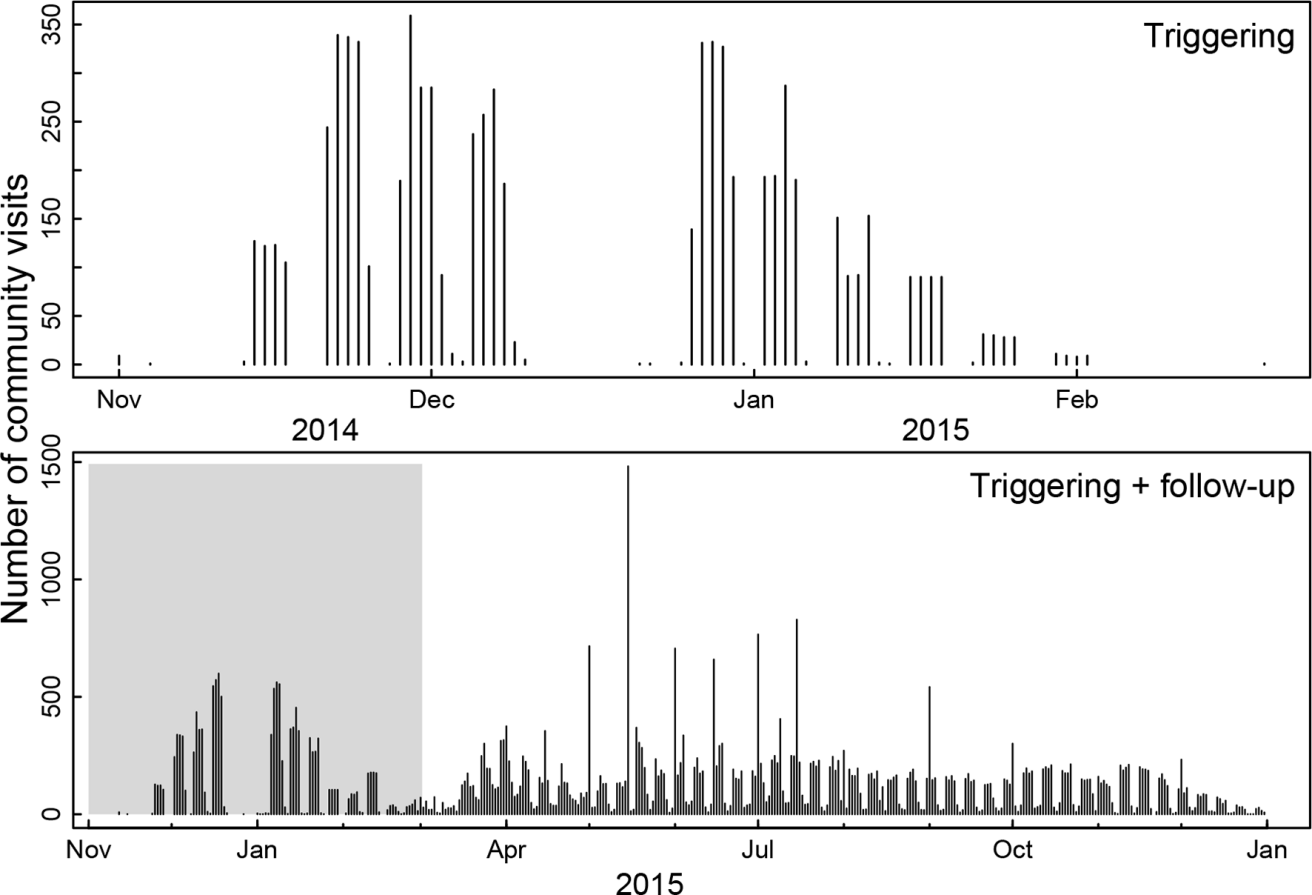
Community visits over time. Figure shows the number of community visits per day for the triggering events (top panel) and for the triggering and follow-up visits (bottom panel).

Regular follow-up visits by mobilisers, combined with support to communities with mobile phone connectivity, enabled the monitoring of progress, listening to emergent needs and changes, linkages to resources and service providers and support for maintenance of agreed actions within communities. Figure 1 shows the number of community visits per day undertaken collectively by mobilisers.

Mobilisers used a standardised form to capture both quantitative and qualitative data gathered from their engagements with communities. Each triggered village was visited on average once every 3 weeks. Quantitative epidemiological data included community surveillance metrics such as the total number of suspected Ebola cases, number of cases referred to a health facility/health alerts within 24 hours of symptom onset, number of survivors, number of suspected deaths, number of safe burials, number of burials conducted by the community and the time elapsed since last suspected case. All quantities were compiled separately for males/females and children/adults. Qualitative items captured commonly expressed concerns, Ebola risk perceptions and narratives on community action plans. These were captured through a set of open-ended questions, including but not limited to the following:

1. What are the most commonly expressed Ebola-related concerns expressed by community members?
2. What were the most commonly asked questions by community members?
3. What did the community initially assess and rank as key risks for contracting Ebola?
4. What action points or bylaws have been developed on Ebola in this community?

Data were collected using paper-based forms across all districts from December 2014 through to September 2015, while a subset of the data from April to September 2015 were collected using a digital system in five active transmission districts (Western Area Urban, Western Area Rural, Port Loko, Kambia, Moyamba and Kono) using Open Data Kit (opendatakit.com). Starting in April 2015, digital data reporting was extended to also document the activities of religious groups and radio stations in the five aforementioned districts. Using these data, weekly community engagement situation reports were developed. These included qualitative reports from communities, actions of national and district pillars, numbers of community visits, mobiliser meetings and alerts, religious leader and radio activities (see ref. 29 and online supplementary information).

## The reach of the CLEA approach

Through the CLEA approach, mobilisers worked with more than 12 000 communities nationally. Mobilisers using the standardised paper forms engaged 2 113 902 community members, of which 50.2% were women while 49.8% were men, and 46% were young people under 18 years while 54% were adults. This number of individual engagements includes multiple interactions with community members who met with mobilisers over multiple visits. During triggering events, the average number of participants per community was 48; in follow-up visits however, the number more than doubled to 113 participants, on average, engaging in community discussions. Distributions of sex and age among participants at triggering versus follow-up visits were not significantly different.

In parallel, using digital reports, mobilisers collected data from both community-level visits and, predominantly in urban areas, house-to-house visits. Mobilisers had 3 129 380 individual engagements with community members across multiple visits. Similar to the visits recorded in the paper-based data, 52% of these were women and 48% men. The average visit consisted of an interaction with 57 community members with most around 25 people, but some as high as hundreds.

**Figure 2:**
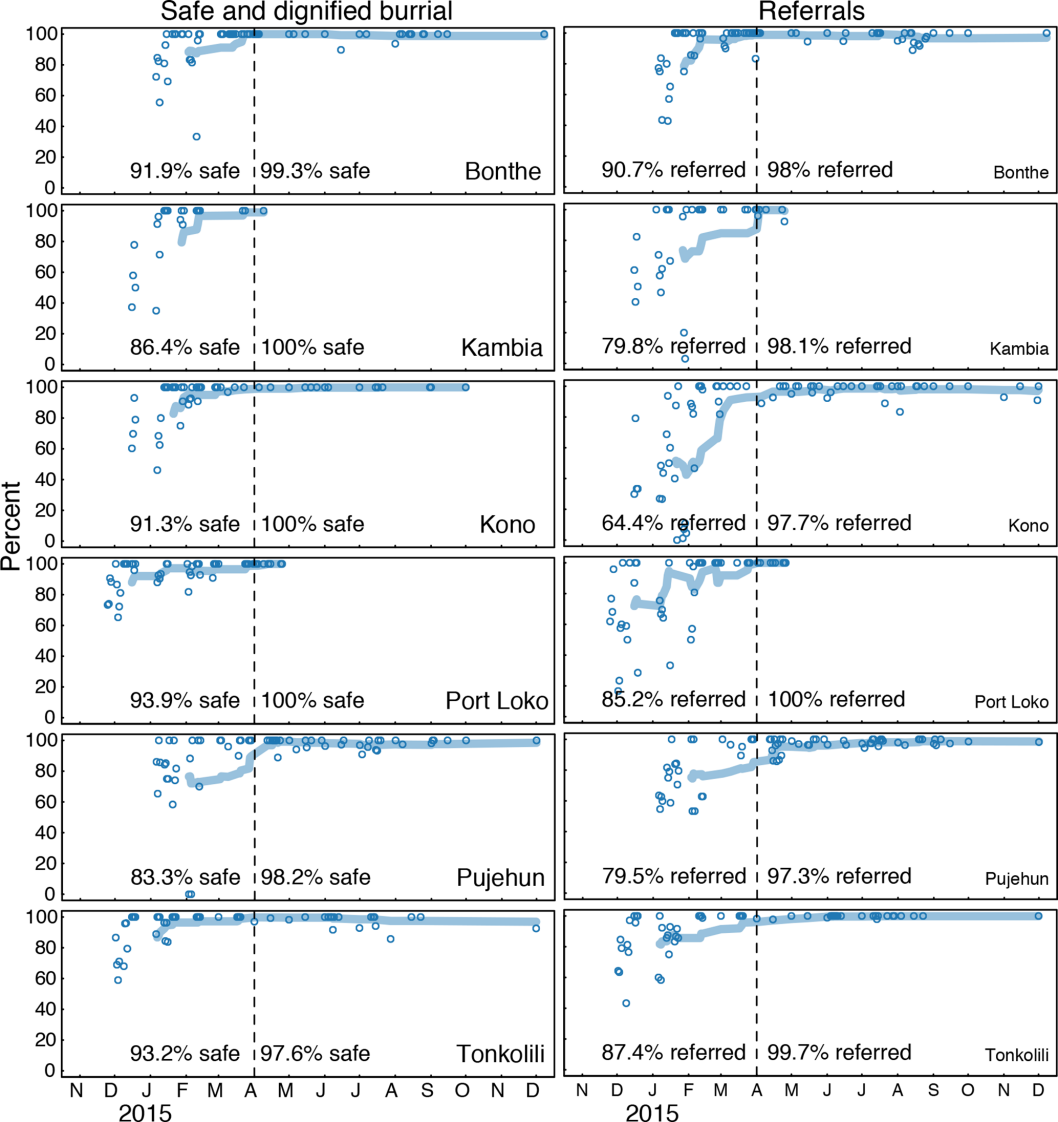
Behavioural impacts of community engagement. Increase in fraction of safe burials following deaths (left) and fraction of cases referred to a health facility with 24 hours (right). We divide the data per district and plot our estimates for per cent of cases referred, and per cent of safe burials following deaths, at different visits. The dotted line shows the transition from period 1 (paper based) and period 2 (digital) which also reflects a point when most communities were already triggered and beginning to undergo follow-up.

**Figure 3:**
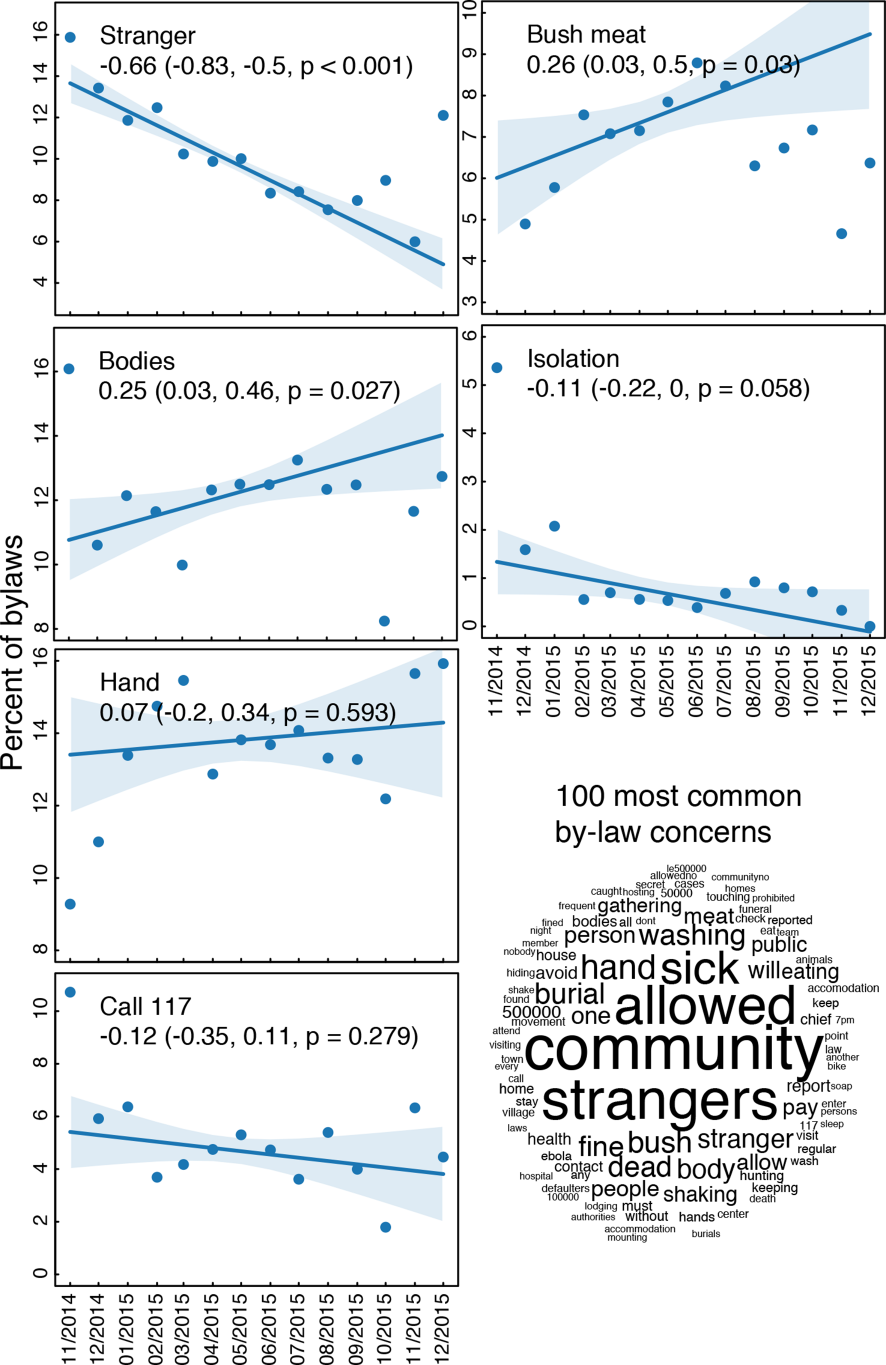
Content of community bylaws. (Left) Frequency of common topics mentioned in community bylaws over time during follow-up visits with regressions weighted by the number of bylaws in the month. (Bottom right) Qualitative representation of the most common concerns and topics in all community bylaws. Numbers refer to the toll-free national alert system (117) and to fines associated with the bylaws (eg, 500 000 SLL ≃ US$60). SLL, Sierra Leonean leone; US$, United States dollar.

The main behavioural outcomes measured during triggering and follow-up visits were (i) timely referrals of sick household members for medical care and (ii) timely requests of safe burials for deceased family members (figure 2). In our analysis, we divided this community surveillance data by district and plotted our estimates for reported per cent of cases referred, and per cent of safe burials following deaths, at different visits. The results indicated an increase over time in the fraction of reported safe burials and fraction of reported cases referred for medical care within 24 hours of the symptom onset. The qualitative data were categorised and themes examined. We then calculated the frequency of common topics mentioned in community bylaws over time with regressions weighted by the number of bylaws in the month (figure 3).

Monitoring data revealed that 100% of communities developed community action plans; action plans contained, on average, three action points. Between April and September 2015, when monitoring was fully operational including through the digital system, mobilisers followed-up on 63 110 cumulative action points. Of these collective action points, 85% were assessed as ‘in-progress’ while 7% were marked as ‘achieved’ and another 7% were ‘not achieved’. Summary statistics from our data on action points and bylaws collected through the paper forms are presented in figure 3.

The data show shifts in action points prioritised and implemented by community members during the intervention period (figure 3). For instance, bylaws around allowing movement restrictions in and out of communities (i.e., community isolation) and consumption of bush meat declined steadily and statistically significantly from November 2014 to December 2015, while bylaws on handling of dead bodies and hand washing increased statistically significantly over this same period. The data also demonstrated relationships between stated bylaws and reported behaviour. For example, early referral was a key action point within the community action plans in the triggered communities. Communities that prioritised early referral as an action item had significantly increased frequency in reports of 24 hours referrals (figure 2).

These data were collected to inform an operational response during an ongoing outbreak. Approval for the SMAC initiative, including community-level data collection, was obtained from the Government of Sierra Leone. Further, the University of Vermont Institutional Review Board (IRB) deemed the work exempt from requiring IRB approval. The data were collected anonymously at the community level. No individual-level data were collected, therefore personal identifiers are not included in the aggregated data set. Mobilisers were trained to obtain verbal informed consent from community members—including community leaders—before completing monitoring data forms during all community visits. Use of the data is subject to terms and conditions outlined in a data-sharing agreement between the Institute for Disease Modeling (in USA) and FOCUS 1000 (in Sierra Leone), the latter being custodian of the SMAC data set.

## The cross-pillar role of the CLEA approach

Monitoring data show that between November 2014 and December 2015 SMAC mobilisers, community champions and religious leaders made more than 4500 alerts to response authorities at district level, through the Ebola 117 hotline as well as directly to district-level alerts desks, which were often manned by SMAC mobilisers.^30^ Although community surveillance was not initially a primary goal of the CLEA approach, it soon became a core component. The incorporation of community surveillance into the SMAC operational model was driven by local needs and was a function of the level of trust established between the mobilisers and the target communities. Mobilisers made an average of 133 community visits per day nationally using paper forms and 151 visits per day nationally using digital reports. Mobilisers received SIM cards and access to free mobile phone calls via a SMAC Closed User Group. All mobilisers were trained in alerts mechanisms within district response authorities (ie, reporting of potential cases or deaths).

These factors essentially established a de facto community surveillance system and closed feedback loop that resulted in mobilisers becoming a primary source of new alerts in some districts. It took some time for response authorities to recognise the need for closer integration of community engagement with other biomedical pillars of the response. These findings indicate the benefits (and potential cost and time savings) of creating strong operational linkages between community-level prevention and other aspects of the response, particularly surveillance. For example, the development of SOPs for community engagement that are integrated with biomedical pillars and afford closer integration is integral within an effective Ebola response.^11 24^

## Large-scale data collection through community engagement

Programmatic data demonstrate that it is possible not only to deliver a large-scale national participatory community engagement intervention in a health emergency context, but also that such participatory methodologies can support the collection of real-time data at scale. The monitoring, follow-up and data collection efforts themselves were able to establish meaningful feedback loops for exchange of information between response authorities and affected communities. This was demonstrated by the increased reliance of district authorities on mobilisers for new alerts, as well as the use of data on community perceptions to inform changes to other response services. Previous studies have shown that digital data collection can be successfully implemented by community health workers with little experience if adequately trained and supervised.^31^ The SMAC experience confirms that such efforts are also possible—and in fact, essential—in the context of a health emergency.

The SMAC model also suggests that behaviour change interventions are most likely to be effective when a combination of communication channels and platforms are used, including community-level interpersonal communication and mass media, and working within an overarching government strategy. This approach is more likely to achieve consistent information and messaging supporting community-led responses that are repeated and reinforced via multiple channels (such as religious leaders and radio), thereby increasing information credibility and reducing confusion caused by mixed messaging.

## Lessons learnt

### 1. Communities are active agents in outbreak response interventions

CLEA demonstrates that communities are not passive recipients of health messages and services. Communities can—and must—be seen as critical agents and equal partners in an emergency response, as they are often best-placed to assess risk and identify mitigation steps in their contexts through collective action planning. Communities are willing to adopt new and sometimes difficult changes to deep-rooted, socio-cultural practices such as funerals and burials, and are capable of arriving at locally-appropriate and acceptable modifications to such practices in response to disease threat.^18^

Analysis of the CLEA approach and other SMAC interventions indicate that it is feasible to support communities to plan for and monitor their own actions in a quantifiable way during an epidemic, provided the right enabling and reinforcing operational structures are in place. Community action plans are more durable and flexible than universal or standardised communications messaging. Participatory action planning methodologies can enable community benchmarking, tracking and adaptation of agreed actions and behaviours over time and as an epidemic evolves. The data on community action plans, as well as the role modeling done by religious leaders as trusted influencers, illustrate how community engagement can facilitate behaviour changes to stop disease spread, especially when targeted in geographical regions of high transmission. Analysis of population-based trends in Sierra Leone show a sixfold increase in intention to wait for safe burial teams and twofold increase in self-reported avoidance of unsafe burials in high transmission regions when comparing before and after the peak of the outbreak.^32^

### 2. Community engagement must be prioritised as a core technical component of epidemic preparedness and operational response

Community engagement, when guided by standardised, but flexible, operational processes can be effectively monitored, sustained and adjusted within the context of an epidemic response. Undertaking community engagement at scale requires clear protocols and guidelines that facilitate a sustained relationship between response authorities, front-line workers and communities. Such protocols include: collection of strong baseline data identifying key behavioural determinants of disease transmission; regular and timely systems for capturing and reporting community monitoring data; systematic and consistent community engagement approaches emphasising two-way communication and feedback loops; established and clear lines of responsibility from response management to front-line mobilisers; iterative mapping of mobilisation activities and systematic identification of emergent issues, cases and trends in localised geographies; continuous supervision and ongoing peer-to-peer support for community mobilisers and front-line support staff; and adequate logistical and communication support.

### 3. Mobilisers and other front-line community workers must be adequately trained, remunerated, supported, and supervised

Community health approaches that depend on what are often termed ‘volunteers’, such as Community Health Workers and other community mobilisers, can be marked by high levels of attrition.^33^ A key characteristic of the SMAC initiative was the low turnover of mobilisers, despite the significant numbers engaged, extended duration of commitment and considerable risks and effort involved.

Since the Sierra Leone Ebola outbreak, the WHO has published the WHO guideline on health policy and system support to optimise community health worker programmes,^34^ and UNICEF has developed Minimum Standards and Indicators for Community Engagement.^6^ SMAC operational policies broadly reflected recommendations contained in these more recent guidelines. These include: clear policies guiding mobiliser selection; appropriate remuneration; adequate pre-training including a 1 week field-based technical training on CLEA participatory approaches (including actual practice with ‘triggering’, as well as curriculum on data collection and use and field safety and security); and supportive supervision, peer-exchange and refresher training by staff supervisors at district level. In addition, mobilisers were provided with insurance coverage, SIM cards and mobile phone credit, along with identifying collaterals (badges, t-shirts, messaging materials). Mobilisers signed contracts and codes of conducts similar to SMAC staff. These structural and operational factors contributed to low mobiliser attrition and providing a mutually accountable framework. Mobilisers and their supervisors were also essential sources of detailed, community-level evaluation of the response.^35^

### 4. Strong field coordination and integrated multiplatform communication strategies enables consistent two-way engagement and avoids community confusion and fatigue

Given its decentralised nature, community engagement in epidemic response can sometimes suffer from poor coordination, resulting in confusing or conflicting messages, as well as inconsistent coverage whereby some communities are neglected or underserved, while others are oversaturated and fatigued. To address this, roles, responsibilities and accountability mechanisms should be clearly outlined for implementing agencies undertaking community engagement activities. Coordination between stakeholders can be greatly advanced through a shared set of protocols or standards guiding practice. Shared standards informing community engagement approaches, communications messaging, training and monitoring systems across implementing agencies can enable consistency, quality interventions from which relevant data can be collected to inform other aspects of the operational response. For example, in Sierra Leone the development of Standard Operating Procedures for Ebola Social Mobilisation, which drew significantly on the CLEA model, significantly improved the operational response in relation to rapid response teams and the district-level coordination.^11^ For CLEA, these operational aspects included how many mobilisers were active, telephone contact details for all mobilisers and community representatives, what locales were being visited on any given day and what data were being collected. To avoid duplication, micro-mapping and sharing of operational activities that was undertaken during the development of the SOPs in Sierra Leone supported greater quality and harmonisation. Current efforts at developing international standards will be invaluable in achieving stronger coordination during future responses.

The SMAC consortium model also suggests that behaviour change interventions are most likely to be effective when a combination of communication channels and platforms are coordinated, combining community-based interpersonal communication such as CLEA with mass media and working in support of government policies. This approach is more likely to achieve rapid behaviour change in an outbreak setting, as consistent information and messaging that support community-led responses are repeated and reinforced via multiple channels, thereby increasing information credibility and reducing confusion caused by mixed messaging.

### 5. Community engagement activities and insights should be integrated within and across other biomedical response pillars and in humanitarian response efforts

Community engagement approaches are focussed on improving the effectiveness of the biomedical response. It spans both demand generation for response services and ensuring that the supply of essential services meets increased demand. This is increasingly relevant as new pharmaceutical tools become available for treating or preventing transmission, yet uncertainty or distrust in communities may avert their uptake. By establishing two-way communication platforms between response actors and communities, community engagement informs decision-making across all aspects of the response. Therefore, response actors should place more emphasis on creating strong functional linkages between community-level prevention and other aspects of the response. Separation of community-based activities from other technical areas that are inherently interconnected risk undermining response effectiveness.

In Sierra Leone, the SOPs were developed by the Social Mobilization Pillar in conjunction with representatives from other response pillars.^11^ The SOPs clearly described how supportive community engagement was to be integrated into technical areas such as surveillance, contact tracing, case management, burials, child protection and psychosocial support.^24^ For SMAC, formalising at response-level already existing operational activities—such as alerts management and surveillance, support burial teams and to families in quarantine—increased transparency and articulated the role of community engagement for all response actors. SOPs and the integration of community engagement should be a priority from the earliest stages of a response, and not phased in once biomedical pillars and protocols are established.

### 6. Real-time community data collection and analysis should be prioritised as essential inputs to inform response decision-making

Community engagement can be monitored at scale in an epidemic response context. Where possible, it should be consistently and rigorously measured during health emergencies of all sizes. Real-time data collection of both behavioural and operational data allows for the findings to inform response decision-making and programming in real time. The granularity of community engagement data collected in real-time at the level of individual communities allows for responses to be localised and responsive to the specific local challenges and opportunities. While existing models for community engagement have developed mechanisms for gathering considerable amounts of social science and behavioural data during the most recent outbreak in the Democratic Republic of the Congo (for example, issues such as rumours and knowledge, attitudes and practices related directly to the disease itself^36^), information collection remains largely unpaired with measurable operational changes or action. It is essential to ensure adequate analysis or integration into other top-level epidemiological data analysis systems, and to overcome the challenge of having this data integrated into the broader response and with epidemiological and health systems data that was experienced in West Africa.^37^

Modelling of the impacts of social interventions on disease transmission in an epidemic is limited by a lack of reliable, at-scale field data collection on social and behavioural indicators, along with indicators that measure the process of community engagement. Tracking a few key community engagement indicators, aligned to a national, government-led monitoring and evaluation framework, should be included in national Situation Reports and mandated for collection and collation by primary implementing agencies.

Placing tools for collection and analysis in the hands of communities, whereby mobilisers or front-line workers are provided with the training, tools and support structures to collect data on community engagement, ensure that the analysis flows back to communities, so they understand how response authorities are using it. Developing a digital data collection system also proved invaluable in ensuring information on behaviours in communities was immediately available. Digital data collection overcomes the limitations of paper-based data collection, including collection, transportation and onerous data entry, as well as inconsistencies in spelling and large degrees of missing information on handwritten forms. Donors and governments should increase investment in digital data collection in order to help generate robust, large-scale, real-time data from communities on health and other social issues affecting quality of life.

### 7. Defining ‘community’ is difficult within the context of epidemic response, but the perfect should not be the enemy of the good

It should be acknowledged that the imperatives of structured community engagement initiatives in humanitarian response can risk undervaluing the complexity of communities and their contexts.^38^ This includes issues related to the heterogeneity of communities, fixed notions of community representation and legitimacy, power dynamics and the role of all stakeholders and the fluidity/contestation of each of these.^39^ They also can underestimate the drivers, structural determinants, biosocial factors and context-specific calculations that drive health-seeking behaviour and decision-making.^40 41^ This being the case, in an emergency the need to achieve scale and two-way communication with rapidity requires not letting the perfect be the enemy of the good.

Despite efforts in design and implementation of the CLEA approach to address issues of participation, inclusion and empowerment, the concept of ‘community’ underpinning the SMAC initiative remained relatively homogeneous. However, for the purpose of rapidly establishing a mechanism for two-way communication at scale, it was found that the framing of ‘community’ focussed on a geographical function, as well as representation of shared values, customs and assets, was effective in garnering sufficient levels of community participation. In Freetown and other urban centres, where ‘communities’ are even more ill-defined, it was found that specific challenges such as lack of community cohesion, high density, informal urban settlement conditions and high mobility required adaptations to the CLEA methodology, including house-to-house visits. There is considerable scope for research on the impacts of community engagement itself on the power dynamics within communities and the effectiveness of models of intervention.

## Limitations

One of the main shortcomings of the SMAC data set is that data from November 2014 through December 2015 were collected through paper reports, while a subset of data from April through September 2015 were collected through digital reports. Direct merging of these data sets proved problematic due to differences in data collection. For instance, some questions that had been open-ended in the paper form entry were included with categorised, multiple-choice type options in the digital form entry. Quantitative epidemiological data were consistently entered across all data collection platforms.

It should be noted that data were self-reported by communities and collected by community mobilisers which may have resulted in positive reporting bias. As such, issues of latent non-compliance cannot be discounted in some communities overreporting based on expectations of Ebola-safe practice.^42^ In addition, it is important to note that the initiation of the SMAC programme occurred as transmission was plateauing in Sierra Leone and as other resources were becoming available, including a more responsive Ebola hotline (117), increased number and professionalism of burial teams and Ebola treatment centres. Moreover, individual level experiences through social learning may have also contributed to the observed improvements in behavioural outcomes.

## Conclusion

The CLEA approach demonstrated that communities are able to plan for and monitor their own actions in a quantifiable way during an epidemic provided the right enabling and reinforcing structures are in place. Evidence for the approach included: strong baseline data identifying key behavioural determinants; systematic and consistent community engagement approaches emphasising two-way communication and feedback loops; regular and timely system for capturing and reporting monitoring data; continuous supervision, top-up training and ongoing peer-to-peer support for community mobilisers; and adequate logistical and communication support, including communications support to communities. Furthermore, the data suggest that communities are capable of engaging in localised surveillance and referral if given the right tools, support and linkages to the formal health structures and systems. Finally, the experience of the SMAC initiative and broader community engagement response in Sierra Leone suggests mechanisms for improving community engagement quality, coordination, integration and monitoring across response actors.
