# Supplementary material for: Community engagement in outbreak response: lessons from the 2014–2016 Ebola outbreak in Sierra Leone
Source: BMJ Glob Health. 2020 Aug 23;5(8):e002145. doi: 10.1136/bmjgh-2019-002145 (PMC7445350; doi:10.1136/bmjgh-2019-002145)
Supplement: Supplementary data [file bmjgh-2019-002145supp001.pdf]

Supplementary Information.

SMAC's Integrated Community Engagement Platform.

The Social Mobilisation Action Consortium (SMAC) was an integrated, large-scale community engagement partnership that served as an integral component to the National Social Mobilization Pillar and the District Ebola Response Centers during the 2014 - 2016 Ebola epidemic in Sierra Leone. SMAC partners included GOAL, Restless Development, FOCUS 1000, BBC Media Action and the US Centers for Disease Control.

This document serves as Supplementary Information to "Community engagement in outbreak response: lessons from the 2014-2016 Ebola outbreak in Sierra Leone" (Bedson et. al.) and describes the core elements of the SMAC initiative, namely:

1. Secretariat and programmatic components;
2. Community-led Ebola Action (CLEA) Mobilizers;
3. Support to Religious Groups;
4. Support to Radio Stations.

These elements provided the basis for a multi-platform, two-way communication mechanism that sought to link communities with response actors and ensure that: i) services reflected community needs; ii) demand was created for Ebola response services.

1. Overview of Social Mobilization Action Consortium Activities.

The SMAC initiative was overseen by a secretariat charged with leading coordination between SMAC partners and the response being led by the Government of Sierra Leone. This included coordination with the National Ebola Response Center (NERC)/Emergency Operations Center (EOC) at national level, District

Ebola Response Centers (DERC) across all districts and Social Mobilization Pillar at both national and district levels. The primary organizational elements of the SMAC initiative included:

- Provision of technical and secretariat services to the National Social Mobilization Pillar and District Social Mobilization Committees;
- Establishment and coordination of an integrated package of community engagement and social mobilization activities including:
    - 2,466 Community Mobilizers implementing CLEA;
    - Support to 42 Radio Stations;
    - Training and support for 2,000 Religious Leaders.
    - Engagement and support of Ebola survivors.
- A coordinating secretariat that worked to ensure integration of all elements of the SMAC consortium, alongside with the Social Mobilization Pillar and other response pillars guiding the biomedical response.
- Real-time data collection and analysis of all interactions undertaken by Mobilizers, complemented by real-time data collection by religious leaders and radio stations.
- Development of weekly national and district level Situation Reports for social mobilization and community engagement which included: i) key challenges; ii) community observations; iii) district level actions/observations; iv) number of triggering and follow-up visits to communities; v) national and district social mobilization pillar updates; vi) death and sick alerts made by Mobilizers.

2. Community-led Ebola Action (CLEA) Mobilizers.

Direct interpersonal community engagement between Mobilizers and communities was facilitated through the Community-led Ebola Action (CLEA) approach, which was designed and implemented by SMAC partners in consultation with staff and volunteers.

The CLEA approach involved assigning Mobilizer pairs to a specific number of communities. In each community, an initial triggering event facilitated by Mobilizers helped community members to undertake their own Ebola risk self-appraisal and analysis. When successful, CLEA triggering created a sense of urgency and a desire to act. Triggering resulted in the development of community action plans and identification of emergent Community Champions. After triggering, regular follow-up visits by Mobilizers supported maintenance of agreed actions within communities. Follow-up sessions included a combination of community discussions and targeted household visits.

The CLEA methodology prioritized community discussion and limited, targeted household engagement over blanket house-to-house (H2H) engagement. However, both SMAC operational and external factors required adaptation of the CLEA approach and explain high household visitation found primarily in Western Area and to some degree in Kambia. In an urban context, adaptations to the CLEA methodology were made due to lack of community cohesion, high density informal urban settlement conditions and high mobility. For example, while Sierra Leone has an overall population density of 79/sq km, while Western Area, comprising the capital city Freetown and surrounding suburbs, has a population density of 1,224/sq km.[i] Large compounds contained a cluster of households/families; and Mobilizers at times supported 'surge' campaign activities directed by the Social Mobilization Pillar, aligning to Social Mobilization Pillar H2H methodologies and working in and around quarantined areas.

Adaptations used in this region included finding alternative champions/influencers in the absence of more traditional leadership structures; developing methodologies for demarcating communities; and using dialogue through household visits.

Key elements of the CLEA approach included:

- Prioritization of recruitment of CHWs, young people with volunteer experience, community members nominated by communities themselves;
- Comprehensive training on the CLEA methodology, interpersonal communication, Ebola transmission, alerts mechanisms, safety and security and data collection;
- Assignment of Mobilizer pairs (one male and one female) to between 6 – 8 communities, to be triggered and follow up in rotation;
- Consultation with District authorities for identification of priority communities and with communities themselves to seek permission for entry and initiating engagement.
- Payment of stipend and reimbursement of travel expenses for all Mobilizers, subject to work agreements and codes of conduct signed by Mobilizers and implementing agencies;
- Weekly meetings of Mobilizers to submit data, receive top-up training and report feedback;
- Submission of monitoring forms signed off by community leaders, including contact details of community representative for verification purposes;
- Identification of a Community Champion in each community, who was provided with a SIM card and mobile phone credit. Community Champions acted as the main point of contact from SMAC Mobilizers.

- Provision of SIM cards and free phone calls via a Closed User Group for Mobilizers.

3. Support to Religious Groups.

There is a mosque and/or church in every community in Sierra Leone, and over 95% of Sierra Leoneans identify as a Muslim or Christian.[ii] SMAC recognized the need for religious leaders to role model safe practices and to provide social support to their peers and congregants, especially in shifting from traditional to Safe and Dignified Medical Burials (SDMB). FOCUS 1000 revitalized the Islamic Action Group (ISLAG) and the Christian Action Group (CHRISTAG) platforms as trusted channels of health communication, formerly leveraged in the late 1980s to achieve universal childhood immunization.[iii] As a result, a high proportion of religious leaders were supported to continuously promote key Ebola messages during Friday and Sunday prayers, while their mosques/churches also provided a range of support to quarantined families and undertook other community outreach events.

A national interfaith taskforce of 25 each of Islamic and Christian Action Group members was established in collaboration with the Sierra Leone Inter-Religious Council. The taskforce identified texts from the Quran and Bible to support the key messages on safe burials, early treatment, and acceptance of survivors and health workers. A total of 1,989 imams and pastors were trained, as well as 4,000 women and youth leaders from 2,000 mosques and churches across the 14 districts in Sierra Leone. A total of 28 faith-based district coordinators – one each of Islamic and Christian Action Group members in each district – were appointed. Similarly, 298 chiefdom coordinators and over 1500 section coordinators were appointed, reaching every section in the country. Such coordinated structure provided a trusted national network for engaging faith-based communities. The trained religious groups – under the leadership of appointed imams/pastors – role modeled preventive behaviors, promoted key practices, and served as trusted messengers in more than 300 radio and television programs.

Activities and reach of religious leaders (April – September 2015)

A total of 4,402 weekly reports were submitted by the targeted religious groups (mosques/churches) in the five districts using the digital data collection system. Of these, 53% were from mosques and 47% from churches. Data revealed that 60% of religious leaders promoted SDMB during sermons, 57% promoted early treatment, and 62% promoted acceptance of survivors and health care workers. Nearly 4 in 10 religious leaders per week participated in SDMBs. Religious leader participation in SDMB varied significantly across districts. It was higher in Kono (45%) and Port Loko (44%) than in Kambia (38%), Moyamba (33%), and Western Area (29%). On average, religious leaders participated in 2 SDMB per week with no significant differences across districts. In addition, 29% of mosques/churches provided support to quarantined homes, whereas 32% carried out other EVD community outreach.

4. Support to Radio Stations.

The KAP-1 survey led by FOCUS 1000 found that radio had by far the widest reach when compared to all other single channels of EVD information, with 88% of respondents reportedly receiving information on EVD through radio.[iv] More precisely, 79% respondents reported listening to the radio daily, while 13% listened weekly. BBC Media Action supported 36 Partner Radio Stations to produce and broadcast approximately 40 hours of Ebola-related programming per week reaching all 14 districts. Partner radio stations produced and broadcast local radio programs, providing district-specific information in local languages to audiences. In addition, BBC Media Action produced two national radio discussion programs and a radio drama. Kick Ebola Live was a two-hour weekly magazine program with interactive SMS component, which was picked up by over 30 radio stations across the country via simulcast. Kick Ebola Nar Salone was a 30-minute pre-recorded show produced weekly and distributed to 36 partner stations

across the country. And the drama entitled "Mr. Plan Plan" encouraged families to face dilemmas, discuss options and develop a plan.[v]

BBC Media Action's partner radio stations collaborated with other SMAC partners to ensure that trusted figures from the community, including representatives from the DERC, religious leaders, Mobilizers and Community Champions, participated in radio discussion programs, providing up-to-date and accurate information and a range of different perspectives.

Activities and reach of partner radio stations (April – September 2015)

SMAC's partner radio stations submitted 120 weekly reports. They collectively produced 189 radio programs (mean of 2 weekly) across the five districts on multiple Ebola-related issues, including prevention, treatment, and transmission. Nearly all radio programs (99.5%) featured a community leader as a live guest or through recorded segments. Collectively, the programs generated 1,619 text messages from listeners. The majority of listeners' text messages (70%) were related to locally driven issues on the effectiveness of the response, the impact of Ebola on education, youth welfare, and employment. In addition, 25% of messages were related to survivors, 22% on burials and 20% on re-infection. Given the small sample size for radio programs, district-level analysis is not appropriate.

SMAC's partner radio stations provided a channel for reaching large proportion of the population with key Ebola information. Radio provided a platform for the participation of trusted messengers such as religious leaders, community champions, and traditional healers as well as survivors and responders to clarify messages and to discuss concerns. Listeners were able to send text messages or call-in to ask questions and share their views. In the earlier stages of the response, rumors and misconceptions were often amplified via low quality radio programming. SMAC supported 36 national and local radio stations through training and mentoring to strengthen the quality of weekly programs in local languages. By

improving the quality of content, SMAC was able to engage audiences and contribute to the accuracy of information disseminated via radio. Using trusted sources on radio, the messages were more likely to resonate with the local populations. This was coupled with more than 189 radio programs wherein community leaders were almost always featured as guests.

[i] United Nations. (2015). *2015 Revision of World Population Prospects.* New York: United Nations.

[ii] Statistics Sierra Leone. (2004). *National Population and Housing Census.* Freetown: Statistics Sierra Leone.

[iii] UNICEF. (1996). *Sustainability of Achievements: Lessons learned from Universal Child Immunization.* New York: UNICEF.

[iv] Jalloh, Mohamed F., et al. (2017). "National survey of Ebola-related knowledge, attitudes and practices before the outbreak peak in Sierra Leone: August 2014." *BMJ global health* 2.4.

[v] BBC Media Action Sierra Leone (2015). '*Mr Plan Plan Episode 1*'. Available at: https://soundcloud.com/bbcmediaaction/mr-plan-plan-drama-episode-1-during-the-ebola-outbreak-in-sierra-leone-liberia-and-guinea
